# Formation of somatosensory detour circuits mediates functional recovery following dorsal column injury

**DOI:** 10.1038/s41598-020-67866-x

**Published:** 2020-07-02

**Authors:** Charlène Granier, Julian Schwarting, Evangelia Fourli, Fabian Laage-Gaupp, Alexandru A. Hennrich, Anja Schmalz, Anne Jacobi, Marta Wesolowski, Karl Klaus Conzelmann, Florence M. Bareyre

**Affiliations:** 1 Institute of Clinical Neuroimmunology, University Hospital, LMU Munich, 81377 Munich, Germany; 2 Biomedical Center Munich (BMC), Faculty of Medicine, LMU Munich, 82152 Planegg-Martinsried, Germany; 3 Graduate School of Systemic Neurosciences, LMU Munich, 82152 Planegg-Martinsried, Germany; 4 Max Von Pettenkofer-Institute, Virology, Faculty of Medicine, and Gene Center, LMU Munich, 80336 Munich, Germany; 5 Munich Cluster of Systems Neurology (SyNergy), 81377 Munich, Germany

**Keywords:** Neuroscience, Regeneration and repair in the nervous system, Spinal cord injury

## Abstract

Anatomically incomplete spinal cord injuries can be followed by functional recovery mediated, in part, by the formation of intraspinal detour circuits. Here, we show that adult mice recover tactile and proprioceptive function following a unilateral dorsal column lesion. We therefore investigated the basis of this recovery and focused on the plasticity of the dorsal column-medial lemniscus pathway. We show that ascending dorsal root ganglion (DRG) axons branch in the spinal grey matter and substantially increase the number of these collaterals following injury. These sensory fibers exhibit synapsin-positive varicosities, indicating their integration into spinal networks. Using a monosynaptic circuit tracing with rabies viruses injected into the cuneate nucleus, we show the presence of spinal cord neurons that provide a detour pathway to the original target area of DRG axons. Notably the number of contacts between DRG collaterals and those spinal neurons increases by more than 300% after injury. We then characterized these interneurons and showed that the lesion triggers a remodeling of the connectivity pattern. Finally, using re-lesion experiments after initial remodeling of connections, we show that these detour circuits are responsible for the recovery of tactile and proprioceptive function. Taken together our study reveals that detour circuits represent a common blueprint for axonal rewiring after injury.

## Introduction

In the motor system it is well-established that the re-wiring of neuronal circuits is the structural basis for functional recovery of the damaged central nervous system (CNS)^1–5^. For this detour circuit to form, corticospinal tract (CST) projection neurons first extend new collaterals into the grey matter, where they make new synaptic contacts with different populations of intraspinal relay neurons. These contacts are subsequently refined and preferentially maintained onto neurons that provide relay connections to the original CST target area^1^. Appropriate target selection of CST collaterals during both, the initial phase of contact formation and the later pruning phase, is required to allow selective compensation of disturbed neuronal pathways without inducing maladaptive responses. The resulting intraspinal detour circuits have been shown to be critical for endogenous functional restoration^1,3,6,7^ as well as for enhanced recovery following neurostimulation and neurorehabilitation^8,9^.

In this study, we asked whether the formation of detour circuits is a unique feature of axonal plasticity in the rodent motor system^1,3,5,8^ or represents a more general blueprint for the remodeling of injured circuits in rodents. To address this question, we studied how ascending somatosensory axonal connections emerging from spinal dorsal root ganglia (DRG) respond to their transection in the mouse spinal cord. Here we focused on proprioceptive afferents, in particular those within the dorsal column-medial lemniscus system, which carries information concerning proprioception and fine mechanical stimuli (touch or vibration) from the spinal cord to the thalamus. Importantly, it has been shown that sensory networks can remodel after injury, in both humans and other primates^10–12^. In particular, following amputation of the hand or dorsal column lesions, there is a reorganization of afferent connections at multiple levels in the spinal cord and the cortex^12–15^. Recovery of hand representation and hand function can be seen months following injury and this recovery is thought to be mediated by spared fibers and a second order spinal cord pathway ascending to the cuneate nucleus via the lateral funiculus^12,14^. While the remodeling of somatosensory circuits is well established in primates, the extent of sensory remodeling following spinal cord injury seems to be variable across species. For example, following dorsal column lesion in rodents, there is a lack of electrophysiological response to tactile hindlimb stimuli in the somatosensory cortex with no apparent adaptive extension of the forelimb tactile responses into the deafferented hindlimb area long after dorsal column injury^16^. However, a progressive expansion of forelimb sensory responses into the deafferented hindlimb area were seen using blood oxygen level-dependent functional magnetic resonance imaging (BOLD-fMRI) and voltage sensitive dyes following larger thoracic spinal cord injury in rats^17,18^. Intraspinal remodeling of synaptic contacts from dorsal root ganglions to dorsal column neurons, supporting proprioceptive functional recovery, has also recently been observed in rats^7^.

In this study, we investigate if and how sensory relay circuits form following a superficial dorsal column lesion in mice. We show that sensory afferents originating from cervical DRGs remodel following spinal cord injury by first increasing the number of axon collaterals that enter the grey matter and second by increasing their contacts onto spinal relay neurons that provide a detour connection to the corresponding brain stem center. We also demonstrate that the targeting of these relay neurons evolves overtime with a preferential increase of connections onto inhibitory interneurons, including parvalbumin positive interneurons known for controlling touch-evoked pain circuitry in the spinal cord^19^. In addition, we show that this DRG connection remodeling mediates functional recovery following injury as such recovery is specifically abolished by secondary lesions ablating the rewired circuits. In short, our work demonstrates that the formation of intraspinal detour circuits occurs not only in the motor system but also in the sensory system, and therefore likely represent a conserved strategy that mediates functional restoration of brain-spinal connectivity.

## Materials and methods

### Mice and anesthetics

All animal procedures were performed according to institutional guidelines and were approved by the Government of Upper Bavaria (animal protocol 55.2-1-54-2532-135-15), and all the methods were performed in accordance with the relevant guidelines and regulations. Mice were maintained on a 12 h light/12 h dark cycle with food and water ad libitum. Adult C57Bl/6j and adult GlyT2-EGFP^20^ mice were used for this study. Unlesioned animals served as controls.

### Viruses design and production

To label ascending DRG axons, we used a recombinant Adeno-associated virus (rAAV) expressing the yellow fluorescent protein (EYFP) following a CAG promoter. To generate rAAV-CAG-EYFP, pAAV-CAG-EYFP was created by inserting Enhanced Yellow Fluorescent Protein (EYFP) from pEYFP-N1 into pAAV-CAG-MCS at the HincII site. pAAV-CAG-ECFP-T2A-G (rAAV-G) was created by inserting ECFP-T2A-G between the EcoRI and HindIII cloning site of pAAV-CAG-MCS. Briefly, ECFP-T2A-G was first PCR-cloned from pAAV-CAG-ECFP onto which the T2A sequence was added using the following forward primer 5′ CGCCCA GAATTC ATG GTG AGC AAG GGC GAG GAG CTG TTC 3′ and reverse primer 5′ CAC GTC ACC GCA TGT TAG AAG ACT TCC TCT GCC CTC TCC GGA TCC CTT GTA CAG CTC GTC CAT GCC GAG AGT 3′. Then, the G envelope protein sequence was PCR-amplified from pCAG-GS-CVS-G using as forward primer GAG GGC AGA GGA AGT CTT CTA ACA TGC GGT GAC GTG GAG GAG AAT CCC GGC CCT ATG GTT CCT CAG GTT CTT TTG TTT GTA and as reverse primer GGG CCC AAG CTT CTA GCT TAC AGT CTG ATC TCA CCT CCA and ligated to the ECFP-T2A fragment. Recombinant AAV chimeric virions containing a 1:1 ratio of AAV1 and AAV2 capsid proteins and the foreign gene were generated as previously described^6,21–23^. For retrograde rabies tracings, we used glycoprotein (G)-deleted SADG-pseudotyped rabies-mCherry (SAD-RABV∆G-mCherry(SADG), which was kindly provided by K.K. Conzelmann (Max von Pettenkofer Institute and Gene Center, Ludwig Maximilians University).

### Surgical procedures

#### Spinal cord injury

Mice were anesthetized with a combination of MMF (Medetomidin 0.5 mg/kg, Orion Pharma; Midazolam 5.0 mg/kg, Ratiopharm; Fentanyl 0.05 mg/kg, B.Braun). Mice were subjected to a laminectomy in order to expose the dorsal region of the spinal cord at cervical level 2 (C2). A unilateral lesion of the superficial dorsal column was made, using fine iridectomy scissors. Care was taken to avoid involving the underlying CST. Following surgery, an analgesic (Metacam, 1 mg/kg, Boehringer Ingelheim) was orally administered. Mice were kept on a heating pad at 38 °C until fully awake.

#### Injections of rAAV and SAD-ΔG-RABV-mCherry

All viral injections were performed 10 days prior to sacrifice. To label DRG axons we pressure injected 0.5 µL of rAAV-EYFP (concentration of 5 × 10^10^ genome copies/mL) into the DRG at cervical level 6 (C6) using a finely pulled glass micropipette (coordinate from DRG surface 0.3 mm depth). The micropipette was left in place for three minutes following the injection to avoid backflow. To label cuneate nucleus projecting neurons, a 1:1 mixture of SAD-RABV∆G-mCherry(SADG) and rAAV-CAG- ECFP-T2A-G (concentration matched to 5 × 10^5^ and 1.5 × 10^10^ genome copies/ml respectively) was injected into the cuneate nucleus. In brief, mice were fixed in the supine position and a fine incision (10 mm long) was made in order to access the medulla oblongata ventrally, holding the trachea to one side using retractors. Once the medulla oblongata was exposed, the union of the two vertebral arteries into the basilar artery was used as the primary reference point for the following injection location. Then, 0.25 µL RABV and rAAV mixture was pressure-injected using a finely pulled glass micropipette (coordinate from union of the two vertebral arteries into the basilar artery: 0.2 mm, 0.9 lateral, 2.0 depth). After completing the injection, the micropipette was left in place for five minutes. Following surgery, an analgesic was orally administered and the mice were kept on a heating pad at 38 °C until fully awake.

### Tissue processing and immunohistochemistry

Mice were deeply anesthetized using isoflurane and perfused transcardially with 4% paraformaldehyde (PFA) in 0.1 M phosphate buffer (PB). Brains, spinal cords and injected DRGs were then microdissected and post fixed overnight in 4% PFA (pH:7.4). The tissue was cryoprotected in 30% sucrose up to 48 h. Using a cryostat, 50 µm sections were cut (longitudinal sections for the anatomical analysis of DRG collaterals, coronal sections for all other analysis). For the analysis of DRG collateral sprouting, the DRG axons localization, the cuneate nucleus projecting neurons localization, the number of contacted neurons and number of contact onto single neurons, a counterstaining was performed with NeuroTrace 435/455 and sections were mounted in Vectashield (Vector Laboratories). For immunohistochemical analysis, we proceeded as follows: Coronal sections, 50 μm, were generated using a cryostat spanning from levels C2 (lesion site) to C6 (DRG injection site). Ten consecutive sections were used for each immunostaining. Tissue derived from C57BL/6 and GlyT2-GFP mice was used to perform parvalbumin (PV) stainings. Sections were first washed three times in Phosphate-buffered saline (1xPBS, pH:7.4) and then incubated for 1 h in a blocking PBS-based solution with 0.5% Triton X-100 and 10% goat-serum (GS). Slices were then incubated at 4 °C in a PBS-based solution containing the primary antibody against PV (Swant PV235 1:2000) 0.3% Triton X-100 and 2.5% GS, for two days. After extensive washing in 1xPBS, sections were incubated overnight at 4 °C with the respective secondary antibody (AF647) and NeuroTrace 435/455 diluted in 1XPBS with 1% GS, washed and mounted with Vectashield (Vector Laboratories). To visualize glutamatergic neurons, antigen retrieval was performed prior to the staining. Because antigen retrieval diminishes the yellow and red fluorescence signals from the injected EYFP and mCherry viruses, antibodies against GFP (Abcam ab13970) and RFP (Abcam ab62351) were used for signal amplification. Heat-mediated antigen retrieval was carried out as follows: Citrate buffer was prepared by adding and mixing 2.94 g of Tri-Sodium citrate (Sigma) with 1 l distilled water for a final concentration of 10 mM and the pH was adjusted to 8.5. Tissue sections were incubated with the heated buffer in a water bath set at 85 °C, for 30 min. Sections were then washed three times in 1 × PBS. Sections were incubated in blocking PBS-based buffer containing 0.5% Triton X-100 and 5% GS. Blocking buffer was replaced with the 1xPBS based solution including 0.3% Triton X-100, 2.5% GS and the primary antibody, anti-glutaminase (Abcam ab93434 1:100), at 4 °C overnight. The second day, slices were washed three times in 1 × PBS and were incubated overnight with the respective secondary antibodies (1:500—goat-anti-chicken Ab 150169 Abcam; goat-anti-rabbit A11012 and A21245 ThermoFischer Scientific; goat-anti-mouse A21236 ThermoFischer Scientific) diluted in 1 × PBS solution with 1% GS. After washing at fifteen-minutes intervals, slices were incubated with primary antibody against RFP (Abcam ab62351, 1:500), and anti-GFP (Abcam ab13970, 1:500) in 1xPBS buffer with 0.3% Triton X-100 and 2.5% GS, overnight. Secondary antibody, AF594, along with the NT-435/455 (N21479 ThermoFischer Scientific), diluted in 1xPBS buffer containing 1% GS, was applied after washing and left overnight for incubation. Finally, slices were washed three times. Tissue was mounted on slides and coverslipped using Vectashield (Vector Laboratories).

### Imaging

*Evaluation of spinal collaterals of DRG ascending fibers and contacts onto cuneate nucleus projecting neurons.* All images were acquired using automated confocal scanning of spinal cord tissue with the FV10-ASW microscopy software on an upright Olympus FV1000 confocal microscope system. Images were obtained using standard filter sets and acquisition settings were kept constant between control and post-injury groups for each experiment. To assess the density of spinal DRG collaterals four sections per animals were randomly chosen and for each sections, four frames were scanned at 20 × magnification (objective: Olympus UPLSAPO 20XO, imaging medium: Olympus IMMOIL-F30CC, NA: 0.85; 640 × 640 pixels, zoom × 1.1, 0.45 µm z-resolution, 16bit). Image fields of view were positioned so that their medial borders aligned with the top of the spinal cord. At this magnification, all of white and gray matter was included, allowing the detection of all spinal DRG collaterals.

*Assessment of DRG tract labeling.* To normalize the number of sprouting fibers to the labeling efficiency, fields of view were centered on the DRG tract in coronal images so that all fluorescently labeled DRG tract fibers as well as a sufficiently large area of the gray matter bordering the CST was included. Images were acquired on an Olympus FV1000 at 20 × magnification (objective: Olympus UPLSAPO 20XO, imaging medium: Olympus IMMOIL-F30CC, NA: 0.85; 640 × 640 pixels, zoom × 2, 0.45 µm z-resolution, 16bit). Images were then processed using ImageJ software to generate maximum intensity projections.

*Evaluation of the DRG collateral distribution in the spinal grey matter*. Axial sections of animals (N = 5 per group) with AAV virus injections into the DRG were used to analyze the location of the DRG boutons. Each 5th section between the lesion at C2 level and the injected DRG at C6 level was evaluated from the consecutively cut spinal cord sections of 50 μm thickness. In total, about 15 sections per animal were used. Under 40 × magnification, the number of boutons was counted under an inverted microscope (Olympus IX71). Subsequently, the mean was determined.

*Evaluation of the contact formation onto different subtypes of interneurons.* As described earlier, DRG axons were labeled with EYFP (AAV-EYFP) and cuneate nucleus-projecting neurons were labeled with RABV-mCherry. Images were acquired as stacks (tile scan acquisition) from 50 μm thick sections, using an upright Leica SP8 WLL confocal microscope. Three sections per staining and per animal were randomly chosen between the C4 and C6 cervical levels and were imaged. Image acquisition settings on the confocal microscope were as follows: (i) scanning conducted in a sequential mode between frames with a × 20/0.75 NA oil-immersion objective, (ii) resolution 2048 × 2048, (iii) frame average 4, (iv) step-size set to 1.5um and (v) zoom at 0.75. NeuroTrace 435/455 and mCherry were scanned together, EGFP with far-red and EYFP alone. To avoid any overlap between the fluorescent signals the excitation and detection wavelengths were carefully set individually for each section and kept constant throughout the experiments.

### Quantifications

All quantifications were performed by an observer blinded with respect to injury status and time points. When data were quantified in the dorsal, intermediate or ventral spinal regions, these quantifications were made according to Rexed laminae: ventral (laminae VIII-IX), intermediate (laminae V-VII) and dorsal (laminae I-IV).

*Quantification of spinal DRG collateral density.* To evaluate the number of collaterals emerging from of DRG axons, longitudinal sections of the spinal cord (50 µm thickness, 4 frames per sections, 4 sections per mice) were acquired with an Olympus FV1000 and the number of DRG collaterals emerging from the main DRG tract was counted. The total number of DRG axons was determined in coronal sections at cervical level C7. The number of collaterals per axon was then calculated.

*Quantification of the DRG collateral distribution.* To evaluate the target areas of DRG collaterals following a sensory lesion, 15 coronal sections spanning the C3 to C6 area of the cervical spinal cord (50 µm thickness, every fifth section) were analyzed under a fluorescence microscope (Olympus IX71) with a × 40/0.65 air objective. The localization of the boutons on these DRG collaterals were categorized as dorsal, intermediate or ventral depending on their localization in the spinal cord. A bouton was defined as a thick varicosity along a comparably thin DRG axon in the cervical spinal grey matter. The number of boutons was expressed as percentage of all DRG axons boutons evaluated in the respective cervical spinal cord.

*Quantification of the localization of cuneate nucleus-projecting neurons.* To evaluate the localization of relay neurons following dorsal column lesion, every coronal sections containing labeled neurons (traced from the cuneate nucleus with the RABV-mCherry) spanning the C3 to C6 area of the cervical spinal cord (50 µm thickness) were analyzed under a fluorescence microscope (Olympus IX71) with a × 10/0.25 air objective. These neurons were categorized as dorsal, intermediate or ventral depending on their localization in the spinal cord. The number of neurons was expressed as a percentage per area of all cuneate nucleus-projecting neurons.

*Quantification of the number of contacted neurons and the number of contacts onto single neurons.* To evaluate the number of contacted neurons and the number of contacts onto single neurons, every coronal sections (50 µm thickness) containing at least one cuneate nucleus-projecting neuron (traced with RABV-mCherry) spanning the C3 to C6 area was acquired with an Olympus FV1000 confocal microscope equipped with standard filter sets and a × 20/0.85 oil immersion objective (zoom 1.5, step size 0.5 µm). All single planes from the image stacks were analyzed in order to assess the presence of boutons (as described above) in close apposition to a cuneate nucleus-projecting neuron (on the neurites or cell body). A contact was defined as the presence of a bouton, a thick varicosity (about three time the diameter of a relatively thick DRG axon^24^) closely apposed to an interneuron’s soma or neurite. To facilitate counting for each subtype in each area (dorsal, intermediate, ventral) the cell counter tool of ImageJ was used to point the contacts on the maximum intensity projections. Both values were normalized to the number of labeled DRG fibers and cuneate nucleus-projecting neurons. The number of cuneate nucleus-projecting neurons was normalized for efficiency by averaging the number of labeled neurons (with the RABV) in three coronal sections rostral to the lesion level (C2). The number of contacted neurons was expressed as percentage of the total number of cuneate nucleus projecting neurons between C3 and C6 of the spinal cord. The number of contacts onto a single neuron was obtained by dividing the total number of contact by the total number of contacted neurons.

*Quantification of the interneurons’ localization and subtype.* For all labeled interneurons, quantification of the location and subtype was done on maximum intensity projections. Image stacks were first loaded on ImageJ and maximum intensity projections were generated. Using Adobe Photoshop and based on the Allen Brain Atlas- Mouse Spinal Cord Reference Atlas, the spinal cord was divided into dorsal, intermediate and ventral areas. The number of each interneuron subtype was counted for each area using the cell counter tool of ImageJ.

### Behavioral analysis

For all behavioral tests, mice underwent three familiarization sessions. The behavior was evaluated at baseline (before lesion) and 2 days after the dorsal column lesion to verify the injury deficits. Thereafter we followed the recovery of function at 7, 14, 21, 42 and 84 days post injury (dpi).

*Forelimb placing response* To assess touch and proprioception, the visual placing test was used^25^. In brief, the mouse is held suspended by the tail next to a table edge. The mouse is slowly advanced toward the edge of the table with its torso. A mouse without injury would extend its upper torso and paws simultaneously to reach the edge of the table. Following familiarization, each mouse is recorded and allowed to reach the edge three times. Each trial is then scored from 0 to 4: (0) once aware of the edge, arches the back and reaches out with both forepaws; (1) reaches with both paws but uninjured paw leads; (2) does not reach occasionally with injured paw; (3) does not reach at all with injured paw; (4) head does not raise and does not reach with both paws. For each mouse, an average of the three scores is calculated.

*“Baton” test* As a second sensory test, the baton test was performed^25^. Briefly, the mouse is held suspended by the tail and allowed to grasp a 5 mm diameter stick with its forelimbs. After the mouse grasps the applicator, it is released while the mouse remains suspended. A mouse without injury would grasp the applicator with both forelimbs. Following familiarization, each mouse is recorded and allowed to grasp the applicator three times. Each trial is then graded from 0 to 4: (0) grasp with both forelimbs; (1) grasp with both forelimbs but loses grasp with injured paw; (2) grasp most of the time with injured paw; (3) does not grasp frequently with injured paw; (4) does not grasp with injured paw at all. For each mouse, an average of the three scores is calculated.

### Statistical evaluation

All results are given as mean ± SEM. GraphPad Prism 7 for Windows (GraphPad software) was used to perform the statistical analysis. Normality was evaluated using the D’Agostino-Pearson omnibus normality test in Prism for all datasets. When data followed a normal distribution we used a two-tailed unpaired t-test or ANOVAs. When data did not distribute normally we used a Mann–Whitney test or Kruskall-Wallis tests. For testing non parametric paired samples (Fig. 5d,e) we used the Wilcoxon’s test. Significance levels are indicated as follows: * or # p < 0.05; ** or ## p < 0.01; *** or ### p < 0.001.

## Results

### Recovery of sensory function following unilateral dorsal column lesion

**Figure 1:**
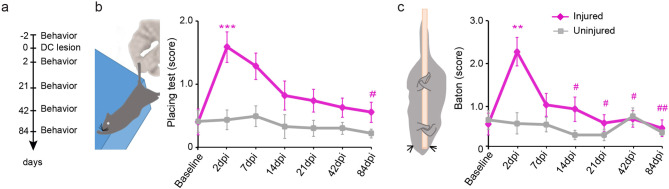
Recovery of sensory function following unilateral dorsal column lesion. (**a**) Experimental setup of the dorsal column lesion paradigm and behavioral testing. (**b**) Schematic drawing of the “placing test” and quantitative analysis of the scores obtained using the placing test at baseline and different time points following dorsal column lesion. (**c**) Schematic drawing of the “baton” test used to evaluate touch/proprioception in mice. Quantitative analysis of the scores obtained using the baton test at baseline and different time points following dorsal column lesion. Datasets were tested first for normality (non-normal distribution) and then analyzed using repeated unparametric ANOVA (Friedmann test) followed by post-hoc multiple comparison Dunn’s tests. “n” equals 12–13 per group. *** p < 0.001: 2 dpi injured vs baseline injured. ** p < 0.01: 2 dpi injured vs baseline injured. ## p < 0.01 84 dpi injured vs 2dpi injured. # p < 0.05: 14, 21, 42, 84 dpi injured vs 2 dpi injured.

We first investigated the recovery of sensory function following incomplete lesions of the spinal cord in mice. To assess this, we performed a unilateral dorsal column lesion at cervical level C2 and monitored the recovery of touch/proprioceptive function using the following two behavioral testing paradigms (Fig. 1a). We first performed the “placing test” to evaluate forelimb coordination, proprioception and tactile input^1,25^. In this test, mice are suspended by the tail and slowly advanced toward a countertop (Fig. 1b). A score is given for the paws ipsilateral to the lesion. Uninjured mice reach out toward the edge of the countertop with the forepaws upon light touch (Fig. 1b). In our study, injured mice showed profound alterations in the test performance of their ipsilateral paws acutely following dorsal column lesion. Over the rest of the study period, mice recovered the ability to perform the test so that by 6 weeks following the lesion the score of injured mice was not significantly different from those of uninjured mice (Fig. 1b; n = 12 mice per group). We also performed the “baton” test, which aims at evaluating tactile input coordination and proprioception^25^. In this test, mice are suspended by the tail and allowed to grasp a thin cotton swab with the paws (Fig. 1c). A score is given for the ipsilateral paws to the lesion. Two days following the dorsal column lesion, the score in the “baton” test was significantly worse in the injured mice (Fig. 1c). Over the rest of the study period, mice recovered steadily and performed similar to uninjured mice from 3 weeks following injury (Fig. 1c; n = 13 mice per group).

### Sprouting of dorsal column-medial lemniscal cuneate fascile pathway

**Figure 2:**
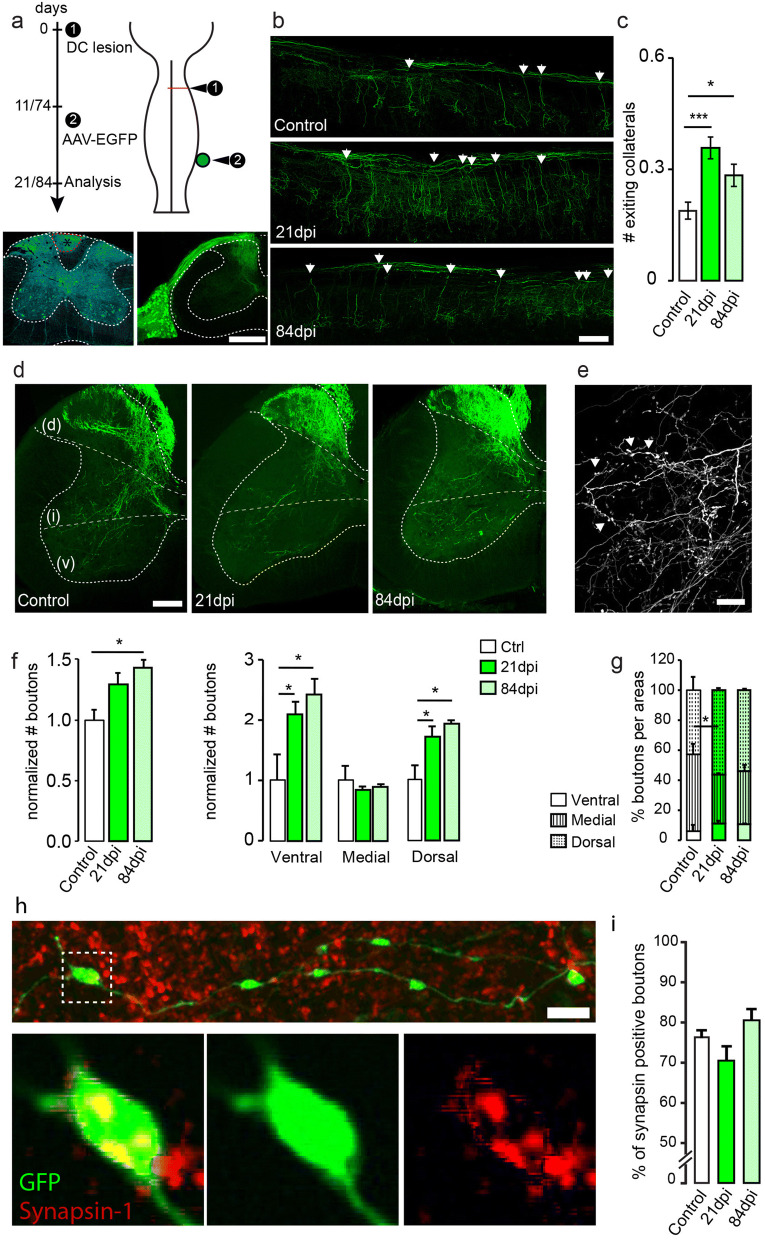
Dorsal column lesion triggers sprouting of DRG collaterals. (**a**) Experimental setup (top) of the dorsal column lesion paradigm and labeling of DRG ascending fibers. Confocal images (bottom) of the center of a representative dorsal column lesion (bottom left) and of a DRG injected with an AAV-EYFP (bottom right) showing the pattern of spinal cord innervation. (**b**) Confocal images of cervical DRG collaterals exiting into the grey matter in unlesioned (control) and lesioned mice at 21 and 84 days post-injury (dpi; arrowheads indicate examples of exiting collaterals; longitudinal view). (**c**) Quantitative analysis of collaterals exiting into the cervical grey matter (***: p = 0.0005; *: p = 0.0183 compared to controls. n = 10–17 mice per group). (**d**) Confocal coronal images of cervical DRG collaterals exiting into the grey matter in unlesioned (control) and lesioned mice at 21 and 84 dpi. Lines on the spinal cord represent the different areas analyzed (dorsal/intermediate and ventral). (**e**) Representative confocal images of the boutons quantified on DRG axon collaterals (image from control animal). (**f**) Quantification of the normalized number of boutons and their change following the lesion (left: general changes; *: p = 0.0285 and right: relative changes in every examined regions *: p = 0.0408 and p = 0.0210 ventral and *: p = 0.0417 and p = 0.0217 dorsal). (**g**) Localization of DRG boutons in the ventral (left panel), intermediate (middle panel) and dorsal (right panel) parts of the cervical spinal cord. Medial: *: p = 0.0386. (**h**) Confocal images of boutons along DRG collaterals double-labeled with synapsin. Bottom pictures are magnifications of the area boxed in the top picture (GFP: green, synapsin-1: red). (**i**) Quantification of the percentage of boutons double-labeled with synpasin-1 in controls and 21 or 84 days following dorsal column lesions. Data analyzed tested for normality (non-normal distribution for c,f,g; normal distribution for i) and analyzed with corresponding tests using Kruskall-Wallis test followed by Dunn’s test in (**c**), (**f**) and (**g**) and tested with a one-way ANOVA followed by post-hoc Dunnett’s’s test for (**i**). In (**i**) n = 471 to 964 counted boutons per group (control: 471; 3 weeks: 567; 12 weeks: 964 boutons). N = 3 mice per group. Scale bars equal 400 µm in (**a**), 200 µm in (**b**, **d**), 50 µm in (**e**) and 5 µm in (**h**). Insets below (**h**) are magnified 4 times from (**h**).

In order to identify the circuit changes that underlie this recovery of sensory function we investigated the anatomical changes of the dorsal column-medial lemniscal cuneate fascile pathway after injury. We first labeled ascending sensory fibers originating from the dorsal root ganglion at C6 anterogradely using a recombinant AAV expressing EYFP (Fig. 2a). We observed that ascending DRG axons branch extensively in the grey matter of the spinal cord in uninjured animals (Fig. 2a,b). Following an unilateral dorsal column lesion, we could observe a significant increase in the number of collaterals emerging from ipsilateral DRG axons caudal to the lesion e.g. between the segments C2 to C6 (Fig. 2b,c). This increased DRG sprouting was initially detected 3 weeks following the injury and the increased collateral density was maintained after completion of the recovery process at 12 weeks following the injury. When we evaluated the repartition of the varicosities on the DRG collaterals we observed that in uninjured mice most of the boutons were located in intermediate and dorsal laminae of the cervical grey matter (Fig. 2d,e). In line with the sprouting of additional DRG collaterals, the number of these boutons was increased at both 3 and 12 weeks after injury (Fig. 2f). Analysis of the repartition of the boutons indicates that most of the newly formed DRG boutons target the ventral and dorsal horns of the spinal cord (Fig. 2f) leading to a relative decrease in the proportion of DRG boutons in the intermediate laminae (Fig. 2g). To confirm that the newly formed varicosities on DRG axons indeed represent pre-synaptic terminals we performed immunostaining for synapsin-1 on a subset of animals. Our results show that across all groups more than 70% of DRG varicosities stain positive for synapsin 1. Notably no differences were observed between varicosities on DRG collaterals in unlesioned mice and those in mice at 3 or 12 weeks after injury (Fig. 2h, i). Taken together, these data show that in response to injury DRG axons sprout new collaterals caudal to the lesion site that establish new synaptic contacts in the cervical grey matter.

### Relay neurons are identified by retrograde trans-synaptic tracing using rabies viruses

**Figure 3:**
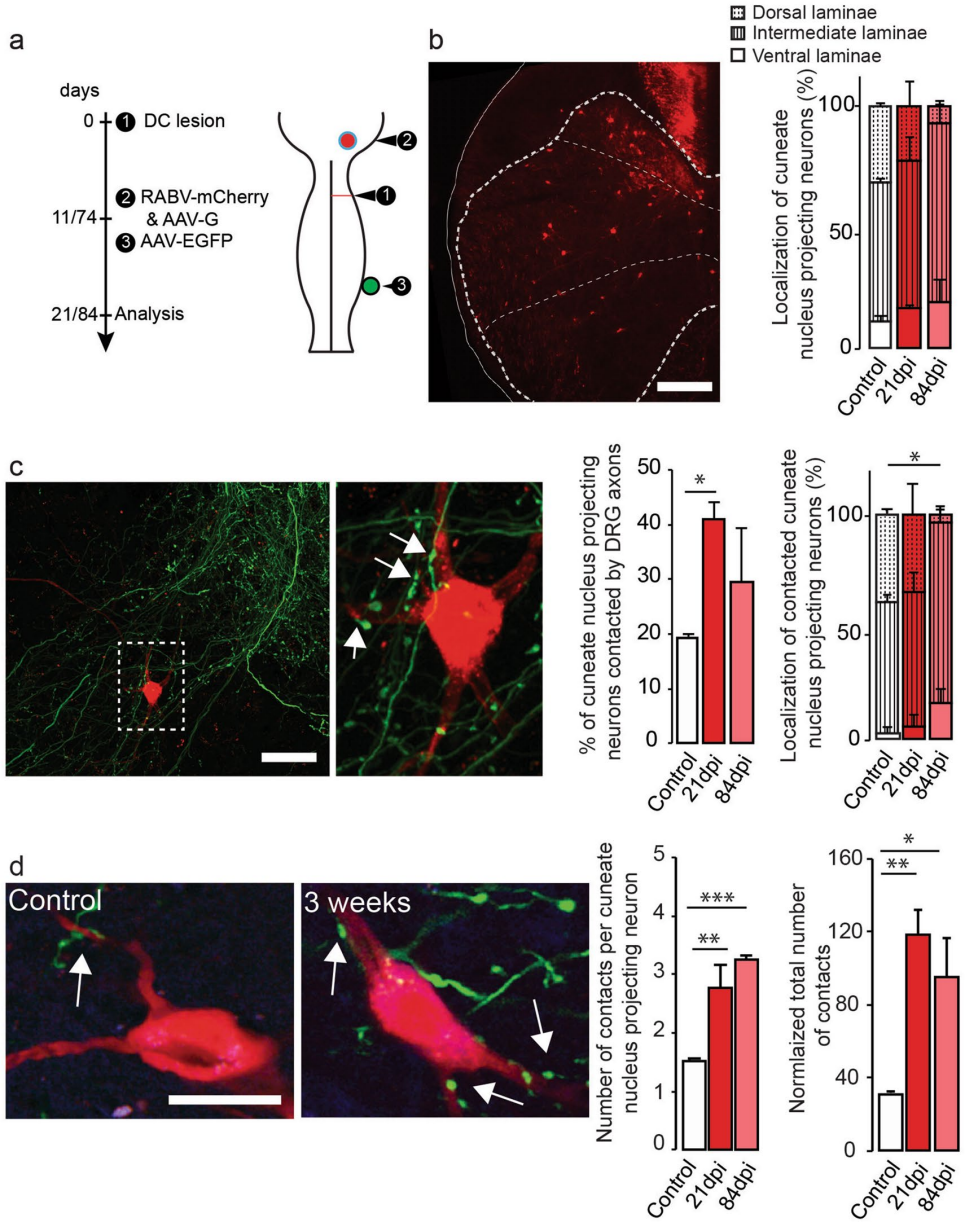
Dorsal column lesion triggers the formation of relays circuits between DRG exiting collaterals and relay neurons originating from the cuneate nucleus. (**a**) Experimental setup of the analysis of the formation of relay circuits. (**b**) Confocal image of cuneate nucleus projecting neurons in the cervical spinal cord following dorsal column lesion and quantification of the percentage of cuneate nucleus projecting neurons contacted by DRG collaterals in unlesioned (control) of lesioned mice at 21 and 84dpi (left panel). Quantification of the localization of cuneate nucleus projecting neurons in unlesioned (control) of lesioned mice at 21 and 84dpi (right panel). (**c**) Representative confocal images of appositions between cuneate nucleus projecting neurons and DRG collaterals in the cervical spinal cord (arrows point to appositions). Quantifications of the % of contacted cuneate nucleus projecting neurons (left) in unlesioned (control) of lesioned mice at 21 and 84dpi (left panel, p = 0.0378) and quantifications of the localization of contacted cuneate nucleus projecting neurons (%, right p = 0.0408 dorsal laminae control vs 12 weeks). (**d**) Representative confocal images of appositions between relay neurons and DRG collaterals and quantifications (left) of the number of contacts per cuneate nucleus projecting neuron (p = 0.0041 control vs 3 weeks and p = 0.00002 control vs 12 weeks). Quantification of the total number of contacts per time points (**: p < 0.01 control vs 3 weeks and *p < 0.05 control vs 12 weeks). Data distribute normally and were analyzed using 1-way ANOVA followed by Dunnett’s multiple comparisons test. Scale bars equal 300 µm in (**b**), 50 µm in ((**c**); right panel is a 3-times magnification of the boxed area) and 15 µm in (**d**).

In order to identify the post-synaptic targets of the newly formed DRG connections, we labeled interneurons in the cervical spinal cord that maintain connectivity to the original target area of the dorsal column-medial lemniscal cuneate fascile pathways after the lesion, and could thus serve as relay neurons for sensory circuits. To this end, we injected a retrograde monosynaptic rabies virus expressing mCherry that transynaptically labels connected neurons^26–28,29^ into the cuneate nucleus (Fig. 3a). Concomitantly we also labeled ascending DRG axons with an adeno-associated virus expressing EYFP injected in the C6 DRG to be able to determine contact formation between the two populations of neurons (Fig. 3a). We performed this experiment in lesioned mice at 3 and 12 weeks following injury and in unlesioned animals. Using this approach, we could show that such cuneate nucleus projecting neurons are present in the unlesioned spinal cord, and indeed can still be detected after lesion indicating that their axons are not transected by the unilateral dorsal column lesion. Cuneate nucleus projecting neurons in the unlesioned and lesioned spinal cord appear to be localized throughout the spinal cord layers with a preference for the intermediate layers (Fig. 3b). Notably, the proportion of such relay neurons that are contacted by DRG collaterals was significantly increased compared to unlesioned mice at 3 weeks following injury (Fig. 3c). We then investigated the location of those neurons contacted by DRG collaterals and found that, in accordance with the overall location of the cuneate nucleus projecting neurons, most contacted relay neurons are located in the intermediate layers of the spinal cord (Fig. 3c). When we evaluated the number of DRG contacts onto a single cuneate nucleus projecting neuron, we found that this number was increased significantly following injury at both 3 and 12 weeks (Fig. 3d). As a result, the total number of DRG contacts onto cuneate projecting neurons is increased by more than 300% at 3 weeks and still by more than 200% at 12 weeks following a unilateral dorsal columns lesion (Fig. 3d). These data highlight that there is a massive and sustained increase in the connectivity between ascending DRG axons and cuneate-projecting interneurons following a spinal lesion, and provide the anatomical correlate for the formation of intraspinal sensory detour circuits.

### Preferential formation of DRG contacts onto inhibitory relay neurons after injury

**Figure 4:**
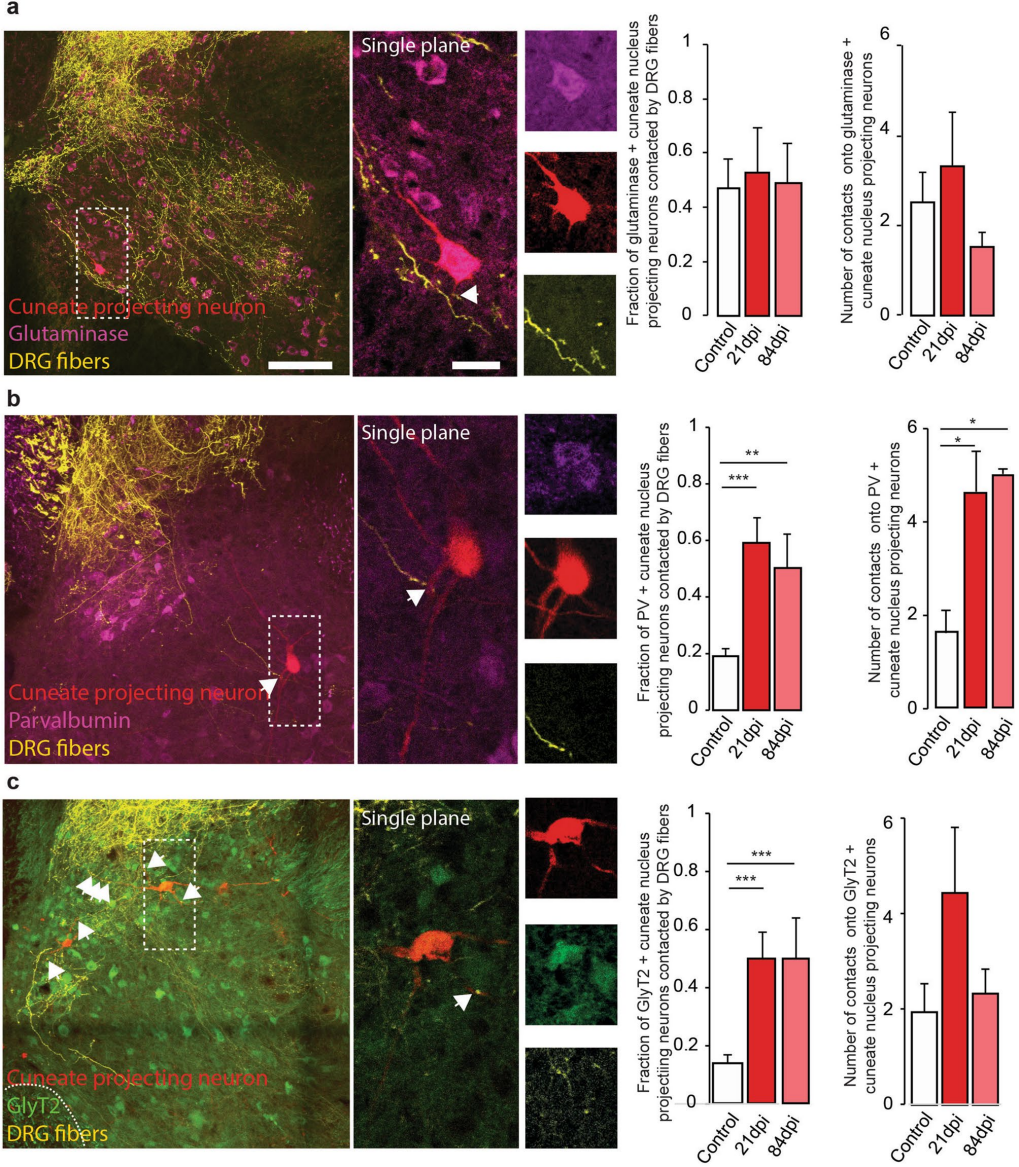
Characterization of the nature of the cuneate nucleus relay projecting relay neurons. (**a**) Confocal images of cuneate nucleus neurons (red) double-labeled with markers for glutaminase (purple). Quantification of the % of cuneate nucleus projecting neurons immunoreactive for glutaminase contacted by DRG fibers (left) and quantification of the number of contacts onto glutaminase positive cuneate projecting neurons (right). (**b**) Confocal images of cuneate nucleus projecting neurons (red) double-labeled with markers for parvalbumin (purple). Quantification of the % of cuneate nucleus projecting neurons immunoreactive for parvalbumin contacted by DRG fibers (left) and quantification of the number of contacts onto parvalbumin positive cuneate projecting neurons (right). ***: p = 0.0003 & **: p = 0.0012 (left) and *: p = 0.0209 & *: p = 0.0125 (right) (**c**) Confocal images of cuneate nucleus projecting neurons (red) and transgenic labeling for glycinergic neurons (GlyT2: green). Quantification of the % of cuneate nucleus projecting neurons immunoreactive for glycine contacted by DRG fibers (left) and quantification of the number of contacts onto glycine positive cuneate projecting neurons (right). ***: p = 0.0003 (left, both comparisons). Areas boxed in the low magnification images are magnified 4 times in the left inset. All DRG fibers appear yellow on the pictures. Data were tested for normality and analyzed then with a one-way ANOVA followed by Dunnett’s post-hoc test. N = 9 sections per group and n = 3 animal per group. Scale bar equal 40 µm in (**a–e**). For insets scale bar equals 10 µm in (**a–e**).

We next aimed to determine which interneuron subtypes make up these sensory relay neurons. To do so we employed the following approaches: we used transgenic mice expressing the green fluorescent protein under the control of promoters such as the glycinergic transporter 2 (GlyT2-GFP) to identify glycinergic neurons^20^. We also used immunohistochemistry to identify glutamatergic neurons by staining for glutaminase. Finally we used immunohistochemistry to identify parvalbumin (PV)-expressing neurons. We first determined the identity of cuneate nucleus projecting neurons in the unlesioned and lesioned spinal cord (Supplementary Fig. 1). Our data show that cuneate projecting neurons often express glutaminase and, in lower proportion, also glycine or parvalbumin. Not surprisingly, the identities of these cuneate nucleus projection neurons were not significantly different between the lesioned and unlesioned spinal cord (Supplementary Fig. 1). We then evaluated the probability of the different cuneate nucleus projecting neuron populations, expressing either glutaminase, glycine or parvalbumin, to be contacted by DRG axons. In unlesioned animals, we found that cuneate nucleus projecting neurons expressing glutaminase have the highest probability to be contacted by DRG collaterals compared to parvalbumin expressing or glycinergic cuneate nucleus projecting neurons (Fig. 4a–c). Interestingly, following injury the proportion of glutaminase expressing cuneate projecting neurons contacted by DRG fibers did not change, while the proportions in both the parvalbumin expressing, and the glycinergic subpopulations, were increased more than two-fold at both 3 and 12 weeks after injury (Fig. 4a–c). In line with these findings, the number of DRG contacts per individual glutaminase-positive cuneate nucleus projecting neurons did not significantly change over time following the lesion (Fig. 4a). In contrast, the number of contacts onto parvalbumin-positive cuneate projecting neurons were increased significantly at both 3 and 12 weeks after lesion (Fig. 4b). Taken together these results suggest that emerging DRG collaterals preferentially contact inhibitory relay neurons. This is an important finding, given that parvalbumin-positive and glycinergic neurons are critical regulators of both spinal and supraspinal sensory processing^19,30–32^.

### Formation of sensory detour circuits is responsible for functional recovery

**Figure 5:**
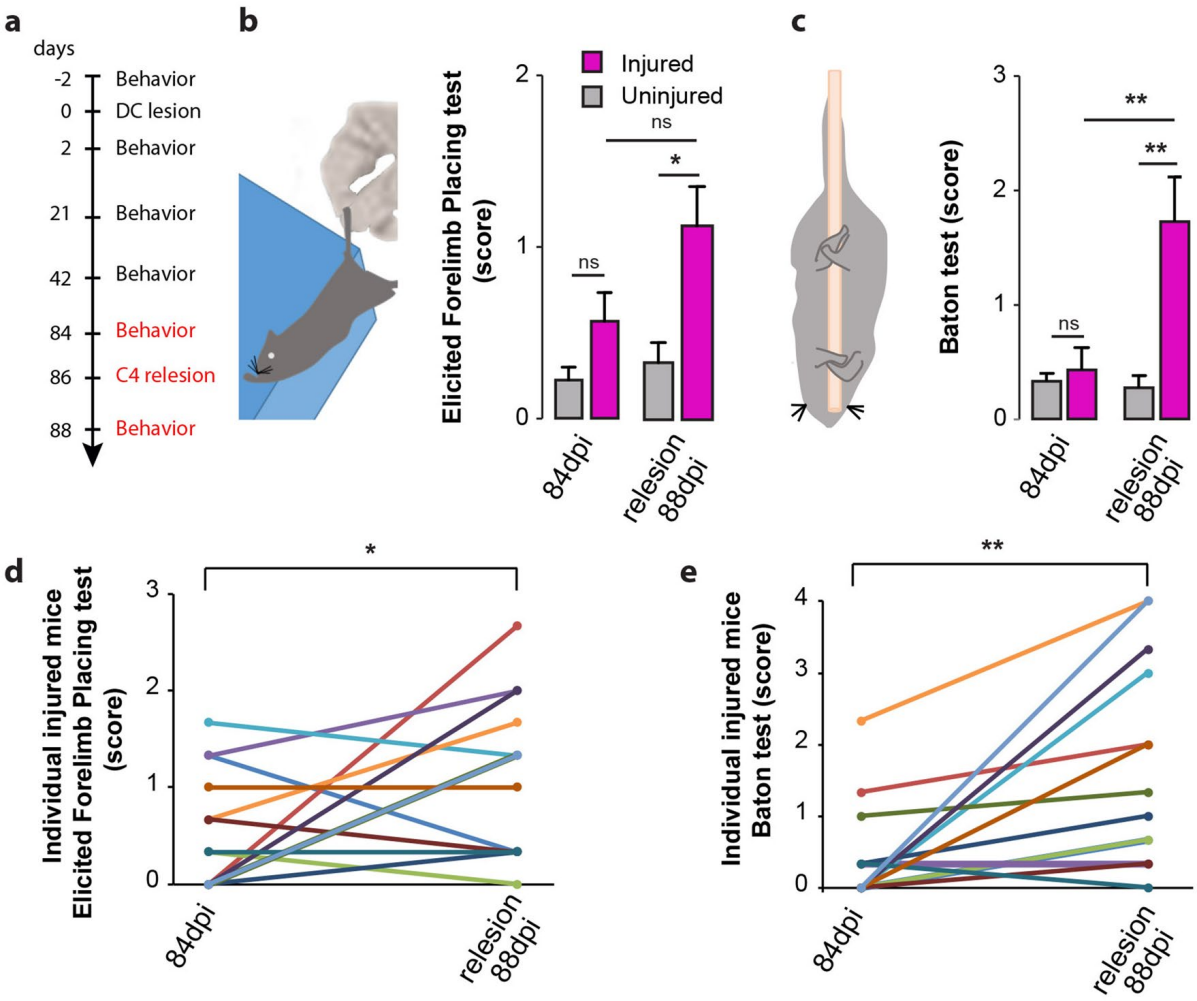
Formation of detour circuits mediates functional recovery. (**a**) Experimental setup of the dorsal column lesion and re-lesion paradigm and behavioral testing. (**b**) Schematic of the “Placing test” and quantitative analysis of the scores obtained with the Placing test at 88 days following dorsal column lesion and following re-lesion experiments at spinal level C4 (p = 0.0248). (**c**) Schematic of the “Baton” test used to evaluate proprioception in mice and quantitative analysis of the scores obtained at the Baton test at 88 days following dorsal column lesion and following re-lesion experiments at spinal level C4 (p = 0.0047). (**d**) Paired scores obtained by individual mice 84 days following dorsal column lesion and re-lesion at C4 in the placing test (p = 0.0488). (**e**) Paired scores obtained by individual mice 88 days following dorsal column lesion and following re-lesion at C4 in the baton test (p = 0.002). Datasets were first tested for normality (non-normal distribution) and then analyzed using a Kruskall-Wallis test followed by post-hoc multiple comparison Dunn’s tests in (**b**) and (**c**) and using unparametric Wilcoxon paired tests in (**d**) and (**e**). “n” equals 12 in (**d**) and 13 in (**e**).

Finally, we wanted to assess whether this reorganization of the dorsal column-medial lemniscal pathway is responsible for the recovery of sensory function following dorsal column lesion. In order to do so, we performed a re-lesion experiment following the 12-week recovery period (Fig. 5a) at the level C4, so that all sprouted collaterals from ascending DRG fibers would be transected and then performed the behavioral tests 88 days following the initial injury. We could show that recovery of function in the elicited forelimb placing test and in the “baton” test was abolished following re-lesion both when we evaluated the performance of all mice grouped together (Fig. 5a–c), as well as when we assessed individual performance trajectories (Fig. 5d, e). These results indicate the formation of sensory detour circuits, initiated by transected DRG axons, is a major contributor to the recovery of proprioceptive function.

## Discussion

The rewiring of spared neural circuits after injury is an important mechanism mediating functional recovery following spinal cord injury^1,3–5^. At the spinal level, most rodent studies so far have focused on the adaptation of motor circuits^1–5,33^, however, recent evidence suggests that somatosensory circuits may be capable of adaptive plasticity as well^7^. Furthermore, the cortical plasticity of sensory representation in response to spinal cord injury is well studied not only in rodents^16–18^ but also in primates^11,13,15^ and humans^10^. Here we focused on revealing the rewiring strategies of sensory circuits by studying the structural adaptation of the ascending medial lemniscal pathway following a cervical dorsal column lesion in mice. Our work demonstrates that remodeling of somatosensory afferents is induced spontaneously following a dorsal column lesion and directly contributes to recovery of sensory function, including proprioception and the sense of touch. We employed the forelimb placing test and the baton test to assess the tactile and proprioceptive capabilities of mice^1,25,34^. A significant recovery process during which mice regained their preinjury status was observed over the course of weeks following the injury, a timeframe that is compatible with the plasticity and adaptation of lesioned or spared sensory circuits^1,7^. This interesting observation is in line with studies in primates, which documented a recovery of cortical hand representation in response to touch of the hand, weeks following dorsal column section ^13,15^ and an involvement of spared and second order spinal cord pathways to the recovery of hand representation in the cortex^12,14^.

Here we observed the formation of new DRG collaterals that project to the cervical spinal grey matter caudal to the lesion site, starting from 3 weeks after injury and persisting for at least 12 weeks. Further analysis showed that the emerging DRG collaterals preferentially target dorsal and ventral layers of the spinal cord, consistent with a previous study that reported the formation of synaptic contacts from DRG axons on a small number of dorsal column neurons following a spinal lesion^7^. Interestingly, we can demonstrate that more than 70% of the boutons are synapsin-positive indicating that they are mature synapses that have integrated into the local spinal circuitry. This is reminiscent of studies performed in primates, in which an increase in the branching of DRG axons in the dorsal horn of the spinal cord after either peripheral amputation of the hand or dorsal root lesion has been reported^13,35^. To study this integration in more detail we used pseudotyped rabies viruses^26–29^ injected into the cuneate nucleus to label, identify and localize the neurons that could relay somatosensory information to the original target area of the dorsal column-medial lemniscal cuneate fascile. We show that these neurons that are mostly located in medial and dorsal laminae of the cervical spinal cord remain connected to the cuneate nucleus after a dorsal column lesion and are thus well suited to serve as relay stations in a spinal detour circuit. Notably, both the percentage of cuneate nucleus projecting neurons that are contacted by DRG collaterals as well as the number of those DRG contacts onto a single such relay neuron are significantly increased following spinal cord injury resulting in a more then 300% increase in total contacts between DRG axons and cuneate nucleus projecting neurons at 3 weeks following injury. Our work ties in with previous observations in primates, in which an increase in the numbers of dorsal horn spinal interneurons projecting to the cuneate nucleus was observed following dorsal column lesion^12,14^. Interestingly, the authors could relate this increase in the number of relay neurons to the recovery of hand use upon tactile stimulation as well as to the re-activation of hand area in the cortex. Here we present a similar reorganization of somatosensory circuits in the injured rodent spinal cord indicating that recovery mechanisms are not only similar between the motor and somatosensory system but may indeed also be conserved across species. Our results are further compatible with a recent study in rats, which demonstrated that in a conditioning lesion paradigm, synaptic contacts onto a restricted number of dorsal horn spinal interneurons can mediate the recovery of proprioceptive function^7^. In our study, we also show that over time, the percentage of contacted neurons appears to decrease while the number of contacts per relay neuron is, if anything, increased. This evolution of the contact pattern is reminiscent of the formation of intraspinal detour circuits in the adult motor system, where contacts are formed within the first 3 weeks after injury and later refined leading to the removal of contacts from some interneurons while the connections to others are strengthened^1,36^. This formation of excessive initial connections that are later pruned is also similar to way circuits are first formed during development, as has been shown, for example, with climbing fibers in the cerebellum or in the visual system^37,38^. When we further characterized the identity of these somatosensory relay neurons we found that most of the cuneate nucleus projection interneurons that are contacted by DRG collaterals in unlesioned mice are excitatory in nature (glutaminase positive), while under these homeostatic conditions only a smaller fraction of inhibitory interneurons expressing either parvalbumin or glycine are contacted by DRG collaterals. Following injury, however the probability of these inhibitory neurons to be contacted by DRG collaterals was strongly increased, while contacts on excitatory interneurons remained largely unchanged. This increased DRG connectivity was observed most prominently for parvalbumin positive neurons, which showed not only an increase in the percentage of contacted neurons, but also a sustained strengthening of the DRG connections onto these neurons. This is of particular interest as parvalbumin positive neurons have been identified as a critical source of inhibition that regulates sensory thresholds by gating mechanical inputs in the dorsal horn^30^, thereby controlling the coordinated activity of neuronal ensembles. This increased connectivity onto spinal inhibitory neurons (glycinergic and parvalbumin positive) can contribute to recovery of somatosensory function, for example by increased inhibition of inhibitory neurons in the cuneate nucleus resulting in enhanced excitatory input to the thalamus. This is in line with the fact that the cuneate nucleus is not only a pre-thalamic relay center but also allows integration of somatosensory information^31^. In a recent study, it was found that glycinergic inhibition provides a critical control of excitability in parvalbumin‐expressing interneurons in the dorsal horn and is therefore also directly modulating spinal sensory processing^32^. Parvalbumin-positive neurons also prevent tactile inputs from activating pain circuits^19^. Maybe the reorganization of contacts onto parvalbumin-positive neurons can also be seen as a strategy to alleviate allodynia following spinal lesions. In primates following sensory lesions, preserved secondary spinal cord afferents to the cuneate nucleus can mediate considerable recovery^14^. By analogy, our study showing a re-routing onto inhibitory spinal neurons can be interpreted as a strategy to activate the cuneate nucleus and trigger functional recovery. This preferential targeting and maintenance of contacts onto “functionally meaningful” relay neurons is another parallel and key characteristic of the formation of corticospinal detour circuits and supports the view that this rewiring of somatosensory circuits is an adaptive strategy to enable the recovery of proprioceptive function. This view is further supported by our re-lesion experiments showing that the recovered sensory function is lost, when the DRG connections caudal to the lesion site are transected, interrupting the formation of remodeled relay circuits.

Taken together our study thus establishes that proprioceptive function of a dorsal column lesion can recover, and that this recovery is, at least in part, mediated by the spontaneous formation of intraspinal detour circuits that re-connect the transected DRG axons with their original target area.

We believe that these findings are of importance for spinal cord injury as in humans such injuries are most often incomplete, sparing some level of tissue that can be harnessed for rewiring of spared circuits^39^. Our study now indicates that common principles guide these rewiring processes both in the motor and somatosensory system and suggests the formation of intraspinal detour circuits as a key structural element of these rewiring processes. As the mechanisms that guide the formation of detour circuits are beginning to be unraveled^24^ and strategies that support their formation are emerging, such circuits might represent a particular promising therapeutic target for the restoration of motor and sensory function in spinal cord injured patients.

## Supplementary information

Supplementary file1

## Data Availability

The datasets generated during the current study are available from the corresponding author on reasonable request.
