# Supplementary material for: Formation of somatosensory detour circuits mediates functional recovery following dorsal column injury
Source: Sci Rep. 2020 Jul 2;10:10953. doi: 10.1038/s41598-020-67866-x (PMC7331809; doi:10.1038/s41598-020-67866-x)
Supplement: Supplementary file 1 — Supplementary file1 [file 41598_2020_67866_MOESM1_ESM.pdf]

Formation of somatosensory detour circuits mediates functional recovery following dorsal column injury.

Charlène Granier[1,2,3,#], Julian Schwarting[1,2,#], Evangelia Fourli[1,2,#], Fabian Laage-Gaupp[1,2], Alexandru A. Hennrich[4], Anja Schmalz[1,2], Anne Jacobi[1,2], Marta Wesolowski[1,2,3], Karl Klaus Conzelmann[4] and Florence M Bareyre[1,2,5]

Supplementary information

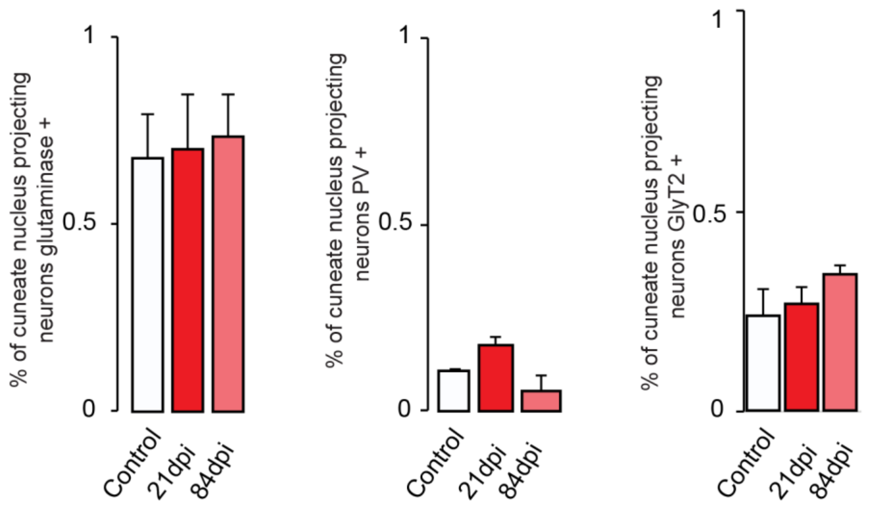

Supplementary Figure 1. Characterization of cuneate nucleus projecting neurons. Graphs indicating the percentage of cuneate nucleus projecting neurons positive for glutaminase, parvalbumin (PV), glycinergic transporter 2 (GlyT2).
